# Embryonic Thermal Manipulation and *in ovo* Gamma-Aminobutyric Acid Supplementation Regulating the Chick Weight and Stress-Related Genes at Hatch

**DOI:** 10.3389/fvets.2021.807450

**Published:** 2022-01-07

**Authors:** Akshat Goel, Chris Major Ncho, Chae-Mi Jeong, Yang-Ho Choi

**Affiliations:** ^1^Department of Animal Science, Gyeongsang National University, Jinju, South Korea; ^2^Institute of Agriculture and Life Sciences, Gyeongsang National University, Jinju, South Korea; ^3^Division of Applied Life Sciences (BK21 Plus Program), Gyeongsang National University, Jinju, South Korea

**Keywords:** embryo manipulation, hatchability characteristics, antioxidant, genes, chickens

## Abstract

Chickens are exposed to numerous types of stress from hatching to shipping, influencing poultry production. Embryonic manipulation may develop resistance against several stressors. This study investigates the effects of thermoneutral temperature (T0; 37.8°C) with no injection (N0) (T0N0), T0 with 0.6 ml of 10% *in ovo* gamma-aminobutyric acid (GABA) supplementation (N1) at 17.5th embryonic day (ED) (T0N1), thermal manipulation (T1) at 39.6°C from the 10th to 18th ED (6 h/day) with N0 (T1N0), and T1 with N1 (T1N1) on hatchability parameters and hepatic expression of stress-related genes in day-old Arbor Acres chicks. The parameters determined were hatchability, body weight (BW), organ weight, hepatic malondialdehyde (MDA), and antioxidant-related gene expression. Percent hatchability was calculated on a fertile egg basis. Growth performance was analyzed using each chick as an experimental unit. Eight birds per group were used for organ weight. Two-way ANOVA was used taking temperature and GABA as the main effect for growth performance and gene expression studies. Analysis was performed using an IBM SPSS statistics software package 25.0 (IBM software, Chicago, IL, USA). Hatchability was similar in all the groups and was slightly lower in the T1N1. Higher BW was recorded in both T1 and N1. Intestinal weight and MDA were higher in T0N1 against T0N0 and T1N1, respectively. The expression of HSP70, HSP90, NOX1, and NOX4 genes was higher and SOD and CAT genes were lower in the T1 group. The present results show that T1 and N1 independently improve the BW of broiler chicks at hatch, but T1 strongly regulates stress-related gene expression and suggests that both T1 and N1 during incubation can improve performance and alleviate stress after hatch.

## Introduction

Chicken producers face numerous stressful conditions that include high ambient temperature, transportation, delayed feeding, infection, noise, and stocking density (1–3). All these conditions collectively or individually may lead to the generation of reactive oxygen species (ROS). Antioxidants react to counter the ROS generation along with the heat shock proteins (HSPs), the marker for identifying stress (1).

Previous studies have shown increment in ROS, antioxidants, HSPs, and MDA under heat stress conditions (4, 5). Evaluation of these genes may help identify the intensity of stress and the preparation of chickens to withstand stress. Furthermore, manipulation of chicken embryos either by tweaking temperature or feeding supplements may develop tolerance to heat stress during the rearing period.

A continuous decrease in the marketable age of broilers has enhanced the importance of embryonic life (6). Embryonic thermal manipulation (ETM), a method of changing the egg's incubation temperature, has been tested to overcome harmful conditions during environmental stress (7). Similarly, *in ovo* feeding, a method of administering nutrients or bioactive substances into the eggs during incubation has been attempted to reduce the dreadful effects of stress associated with transportation and delayed feeding (2, 8). Although embryonic manipulation has been performed for a few limited aspects, it also has the potential to reduce the negative effects of other stressful conditions. Few studies have shown that ETM can be effective to improve growth performance by increasing body weight at market age (6, 9). Most of the studies evaluated the effect of ETM during the later stages of broilers' life during the rearing phase (7, 10). However, scanty information is available on the role of ETM in modulating hatchability performance and on its mechanism to develop stress tolerance in chicks at hatch.

Gamma-aminobutyric acid (GABA) as a feed additive or feeding *in ovo* has shown some potential to overcome stress in chickens (11–14). GABA is a four-carbon non-protein amino acid that acts as an inhibitory neurotransmitter in the central nervous system (15). Other than controlling neuronal excitability, the receptors of GABA were also observed in the liver, pancreas, and kidney (16). Previous studies have reported beneficial effects of dietary supplementation of GABA in laying hens and broiler chickens reared during summer conditions or under heat stress (17–19). Although *in ovo* feeding has been previously shown to be effective in increasing chicken performance and immune functions (8, 20), it is not yet clear whether or not *in ovo* administration of GABA is beneficial for improving the hatchability status of chicks. Moreover, it is uncertain whether or not ETM along with *in ovo* GABA injections could synergistically influence hatchability performances without any adverse effects.

The present study was designed to investigate the effect of ETM, GABA supplementation, or their combination on hatching performance and expression of stress-related genes in newly hatched chicks.

## Materials and Methods

All the experimental procedures for this study were approved by the Institutional Animal Care and Use Committee of Gyeongsang National University GNU-200916-C0058.

### Incubation and *in ovo* Feeding Procedure

**Figure 1:**
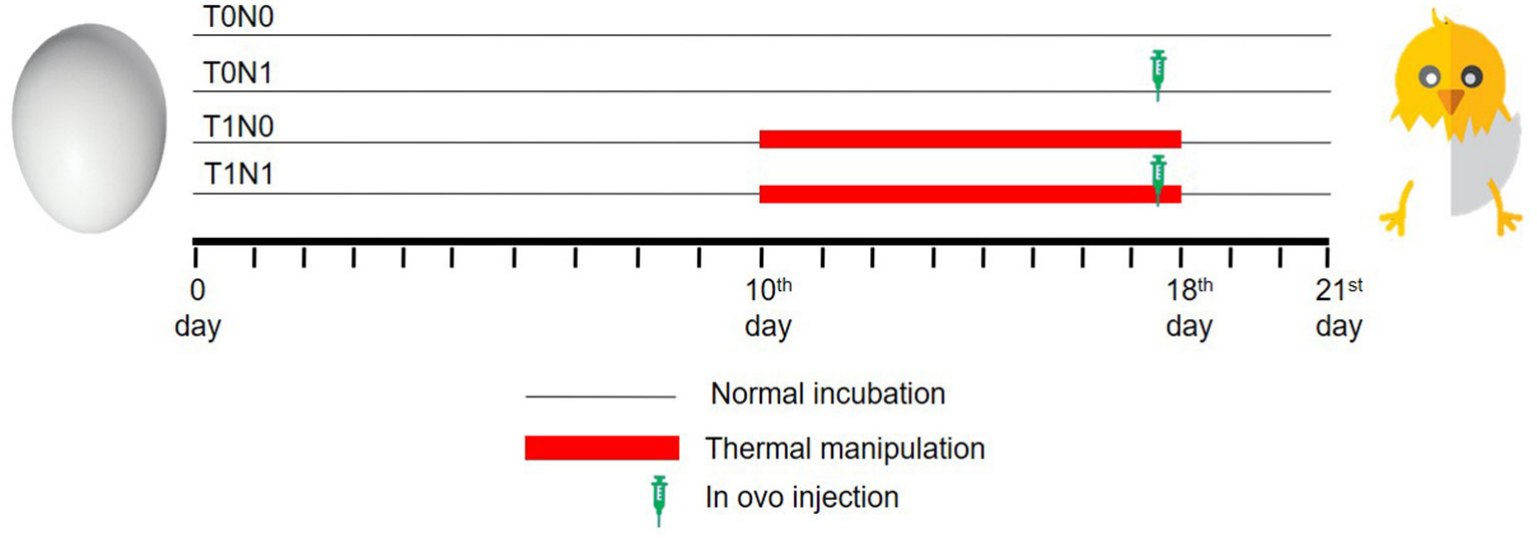
Experimental design showing thermal manipulation duration and *in ovo* feeding time during incubation. T0: Normal temperature; T1: thermal manipulation; N0: No *in ovo* injection; N1: *in ovo* GABA injection.

A total of 280 eggs, obtained from a local Arbor Acres breeder farm (Hapcheon, Korea), were used for this study. The age of the broiler breeder birds was around 30 weeks. Each egg was weighed using weighing balance (PB4002-S/FACT, Mettler Toledo, Switzerland) with 0.01 g accuracy, marked, and then incubated in two incubators (Maru 190, Rcom, South Korea) at 37.8°C and 56% relative humidity (RH), and turned automatically once an hour. At 10th embryonic day, the eggs were candled individually (Candler 200, Rcom, South Korea) and those without embryos were removed from the incubator. The remaining eggs were randomly assigned to one of four groups, with similar mean egg weight (59.6 ± 0.16 g) and number. Two groups were used for normal temperature (T0) at 37.8°C and the other two for thermal manipulation (T1) at 39.6°C. All the eggs were kept in the incubator that was maintained at 37.8°C and only eggs belonging to T1 group were shifted to the incubator maintained at 39.6°C for 6 h daily from 10:00 a.m. to 4:00 p.m. For each treatment group, one was untreated (N0) and the other was administered intraovally with 10% GABA (N1). So, there were 4 treatment groups: T0N0; T0N1; T1N0; and T1N1. After candling, T0N0 had 62 eggs while the rest three treatment groups had 63 eggs each. The eggs for T0 and T1 were kept in two separate incubators (Figure 1). The only difference between the two incubators was that the temperature of the incubator with T1 eggs was increased to 39.6°C from 10th ED to 18th ED for 6 h daily. The selection of GABA dosage for *in ovo* injections was based on our previous studies (12). In the same study, there were no differences in hatchability characteristics between the *in ovo* water-injected group and the non-injected control. So, we did not include a water-injected group in the current study. The selection of temperature (39.6°C) and time (6 h a day) for ETM during incubation was done based on a previous study (21).

**Figure 2:**
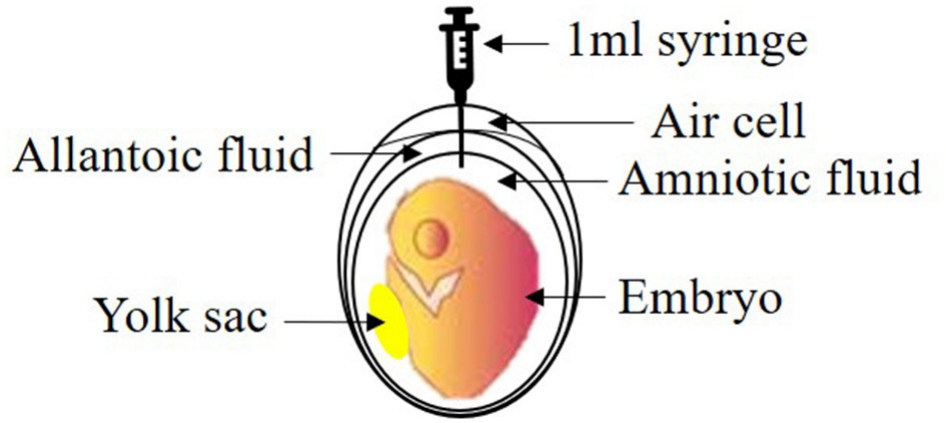
Estimated injection site in the chicken egg at 17.5th ED.

Three hours before injection, 10% GABA (Cat. No. A2129, Sigma-Aldrich Inc., St. Louis, MO, USA) solution was prepared in distilled water and stored at 30°C until use. The selection of day for *in ovo* injection was based on a previous study (22). At 17.5th ED, the eggs for T0 and T1 received *in ovo* injections of 0.6 ml of 10% GABA solution from the blunt end of the egg (Figure 1). At first, *in ovo* injection was carried out in T0N1, followed by T1N1. The time taken for *in ovo* injection procedure was minimized to 5–7 min for each tray. The un-injected control eggs were also removed from the incubator for the same time period to provide similar conditions to all the treatments. All of the eggs were surface-sterilized using 70% alcohol before drilling the broad end (Figure 2). A 1 ml sterile hypodermic syringe with a 1-inch 23-gauge needle (Kovax-syringe® Korea Vaccine Co., Ltd. Seoul, Korea) was used for each *in ovo* injection to target the amniotic fluid. The eggs were then sealed with surgical tape (3M™ Micropore™, Saint Paul, Minnesota) and returned to the incubators. The eggs then remained in the incubator until the day of hatch. During the last 3 days of the incubation, both incubators were kept at 36.8°C temperature and 70% RH.

### Tissues Sampling

At the time of hatch, each chick was individually weighed, and eight chicks from each group were randomly chosen, and humanly euthanized using carbon dioxide. The weight of selected organs was recorded and expressed as a percent to BW. Liver tissues (*n* = 6) were collected and snap-frozen in liquid nitrogen for the determination of malondialdehyde (MDA) and gene expression. The selection of the liver tissue for evaluating the stress and antioxidant-related genes was based on the previous studies (7, 12).

MDA, a marker of oxidative stress, was measured by using the methods (23, 24) with some modifications. Briefly, 100 mg liver samples were homogenized in 1 ml of cold 100 mM Tris buffer, pH 7.4 (1:10 w/v), and centrifuged at 1000 × g for 10 min. After separation, one part of the supernatant was added to one part of 40% trichloroacetic acid (TCA) and 2 parts of 0.67% thiobarbituric acid (TBA), which was placed at 95°C for 45 min on a water bath. The samples were then removed from the hot water bath and placed on ice-cold water and then centrifuged at 9950 × g for 30 s to record supernatant absorbance at 530 nm.

### Real-Time PCR for mRNA Quantification

RNA was extracted from 50 mg liver tissue using Trizol™ reagent (Thermo Fisher Scientific, Waltham, MA, USA) following the manufacturer's instructions. The concentrations and purities of RNA samples were determined spectrophotometrically using a Nanodrop (Thermo Scientific, Waltham, MA, USA). Subsequently, 2 μg of each RNA sample were used for the synthesis of cDNA using the PrimeScript™ first-strand cDNA synthesis kit (Takara, Tokyo, Japan) following the manufacturer's instructions. The cDNA thus produced was used as a template for the amplification of different genes using real-time polymerase chain reaction (RT-PCR).

The amplification of stress and antioxidant-related genes was carried out in StepOnePlus™ real-time PCR systems (Life Technologies, CA, USA). Each reaction consists of a 20 μl containing 10 μl Power SYBRTM green PCR master mix (Life Technologies, CA, USA) and 10 μl of 10 pmol concentration of forward and reverse primers specific for each gene and cDNA prepared in nuclease-free water. The sequences of the primers are presented in Table 1. The PCR cycling program was set to 95°C for 10 min initially and then 40 cycles of 95°C for 15 s and 60°C for 1 min. Execution of melting curves was performed to ensure a single specific PCR product for each gene. Relative expression of various genes was analyzed using the 2^−ΔΔCt^ method and was then normalized using the relative abundance of the control. GAPDH was used as a housekeeping gene based on the previous studies (9, 12, 27).

**Table 1:** Primer sequences used to evaluate the hepatic gene expression on the day of hatch.

**Gene 1**	**Sequence**	**Accession number**	**References**
HSP70	F: GCTGAACAAGAGCATCAATCCA	AY143693.1	(25)
	R: CAGGAGCAGATCTTGCACATTT		
HSP90	F: CCCGAGCAAGCTGGATTCT	NM_001109785	(25)
	R: GGTCATCCCTATGCCGGTATC		
NOX1	F: GCGAAGACGTGTTCCTGTAT	NM_0011018301	(5)
	R: GAACCTGTACCAGATGGACTTC		
NOX4	F: CCTCTGTGCTTGTACTGTGTAG	NM_001101829.1	(5)
	R: GACATTGGAGGGATGGCTTAT		
SOD	F: AGGGGGTCATCCACTTCC	NM_205064.1	(26)
	R: CCCATTTGTGTTGTCTCCAA		
CAT	F: ACCAAGTACTGCAAGGCGAA	NM_001031215.1	(26)
	R: TGAGGGTTCCTCTTCTGGCT		
GAPDH	F: TTGGCATTGTGGAGGGTCTTA	NM_204305.1	(25)
	R: GTGGACGCTGGGATGATGTT		

*HSP70, heat shock protein 70; HSP90, heat shock protein 90; NOX1, nicotinamide adenine dinucleotide phosphate oxidase 1; NOX4, nicotinamide adenine dinucleotide phosphate oxidase 4; SOD, Superoxide dismutase; CAT, Catalase; GAPDH, glyceraldehyde-3-phosphate dehydrogenase*.

### Statistical Analysis

Data were analyzed using an IBM SPSS Statistics package 25.0 (IBM software, Chicago, IL, USA). Percent hatchability was calculated based on the number of chicks hatched to the fertile eggs following the previous studies (9). Each bird acted as an experimental unit for BW at hatch. Eight birds from each group were used to collect the organ weight. All the data were expressed as mean ± standard error of the mean (SEM). Shapiro Wilk and Levene's-tests were used to assess the normality of distribution and equality of variances. A two-way ANOVA was used taking temperature and GABA as the main effect. A Duncan's multiple range test was used if an interaction existed. Parametric differences were considered statistically significant at *p* < 0.05.

## Results

Table 2 shows the effects of embryonic T1 and N1 on hatchability parameters. Hatching rates were similar in all groups except that the T1N1 group was 6-8% lower than the other groups.

**Table 2:** Effects of temperature manipulation and GABA supplementation during embryogenesis on hatchability parameters in broiler hatchlings.

**Treatments**		**Incubated eggs (*n*)**	**Initial egg weight (g)**	**Healthy chicks (*n*)**	**Hatchability (%)**
**Incubation temperature**	***In ovo* GABA**				
Normal temperature (T0)	0 (N0)	62	59.6 ± 0.36	45	75.8
	10% (N1)	63	59.6 ± 0.30	47	76.2
Thermal manipulation (T1)	0 (N0)	63	59.6 ± 0.35	48	77.8
	10% (N1)	63	59.6 ± 0.31	42	69.8

*Initial egg weight data show mean ± SEM*.

Both T1 and N1 significantly increased BW at hatch (*p* < 0.01), but there was no significant interaction between the two (Table 3).

**Table 3:** Effects of temperature manipulation and GABA supplementation during embryogenesis on body weight (g) at hatch.

**Treatments**		**Body weight (g)**
**Incubation temperature**	***In ovo* GABA**	
Normal temperature (T0)	0 (N0)	37.51 ± 0.38
	10% (N1)	38.63 ± 0.43
Thermal manipulation (T1)	0 (N0)	38.89 ± 0.23
	10% (N1)	39.65 ± 0.35
**Main effects**		
Temperature	Normal temperature (T0)	38.08 ± 0.29
	Thermal manipulation (T1)	39.24 ± 0.21
GABA	0 (N0)	38.22 ± 0.23
	10% (N1)	39.11 ± 0.28
***p*** **-values**		
Temperature		0.001
GABA		0.009
Temperature × GABA		0.621

*Data show mean ± SEM*.

T1 and N1 had no significant effect on relative organ weight at hatch. However, intestine weight percent to BW had a significant interaction (p = 0.028) and was higher in T1N1 (Table 4).

**Table 4:** Effects of temperature manipulation and GABA supplementation during embryogenesis on relative organ weights (percent to body weight) at hatch.

**Treatments**		**Liver (%)**	**Gizzard (%)**	**Proventriculus (%)**	**Heart (%)**	**Intestine (%)**	**Yolk remnant (%)**
**Incubation temperature**	***In ovo* GABA**						
Normal temperature (T0)	0 (N0)	2.36 ± 0.12	5.23 ± 0.17	0.94 ± 0.08	0.69 ± 0.04	4.74^ab^ ± 0.38	8.61 ± 1.16
	10% (N1)	2.52 ± 0.10	4.72 ± 0.13	0.86 ± 0.04	0.76 ± 0.03	5.53^b^ ± 0.30	9.66 ± 0.64
Thermal manipulation (T1)	0 (N0)	2.40 ± 0.07	5.03 ± 0.18	0.77 ± 0.04	0.78 ± 0.07	4.69^ab^ ± 0.23	10.07 ± 1.16
	10% (N1)	2.48 ± 0.09	5.26 ± 0.16	0.88 ± 0.05	0.76 ± 0.04	4.43^a^ ± 0.24	10.37 ± 0.46
**Main effects**							
Temperature	Normal temperature (T0)	2.44 ± 0.08	4.97 ± 0.13	0.90 ± 0.04	0.73 ± 0.03	5.08 ± 0.27	9.13 ± 0.65
	Thermal manipulation (T1)	2.44 ± 0.06	5.15 ± 0.12	0.83 ± 0.03	0.77 ± 0.04	4.56 ± 0.17	10.23 ± 0.57
GABA	0 (N0)	2.38 ± 0.07	5.13 ± 0.12	0.86 ± 0.05	0.74 ± 0.04	4.71 ± 0.22	9.29 ± 0.82
	10% (N1)	2.50 ± 0.06	4.99 ± 0.13	0.87 ± 0.03	0.76 ± 0.03	4.89 ± 0.24	10.01 ± 0.39
***p*** **-values**							
Temperature		0.442	0.223	0.826	0.143	0.101	0.078
GABA		0.54	0.517	0.922	0.543	0.402	0.308
Temperature × GABA		0.679	0.129	0.114	0.32	0.028	0.5

*Data show mean ± SEM (n = 8)*.

a,b *: different letters indicate significant differences (P < 0.05)*.

**Figure 3:**
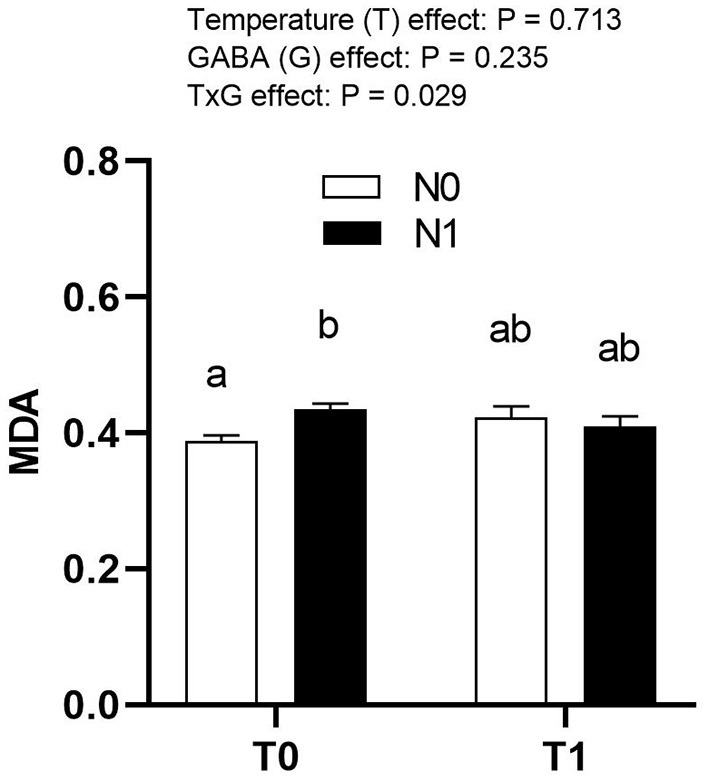
Effects of thermal manipulation (TM) and GABA supplementation (GABA) during embryogenesis on hepatic MDA content (nmol/100 mg) at hatch. Data show mean ± SEM (*n* = 6). T0: Normal temperature; T1: thermal manipulation; N0: No *in ovo* injection; N1: *in ovo* GABA injection. ^a,b^: different letters indicate significant differences (*P* < 0.05).

Both T1 and N1 did not have a significant effect on hepatic MDA at hatch (Figure 3). However, there was a significant interaction between temperature and GABA (*p* = 0.029), as MDA was higher in T1N1 compared to T0N0.

**Figure 4:**
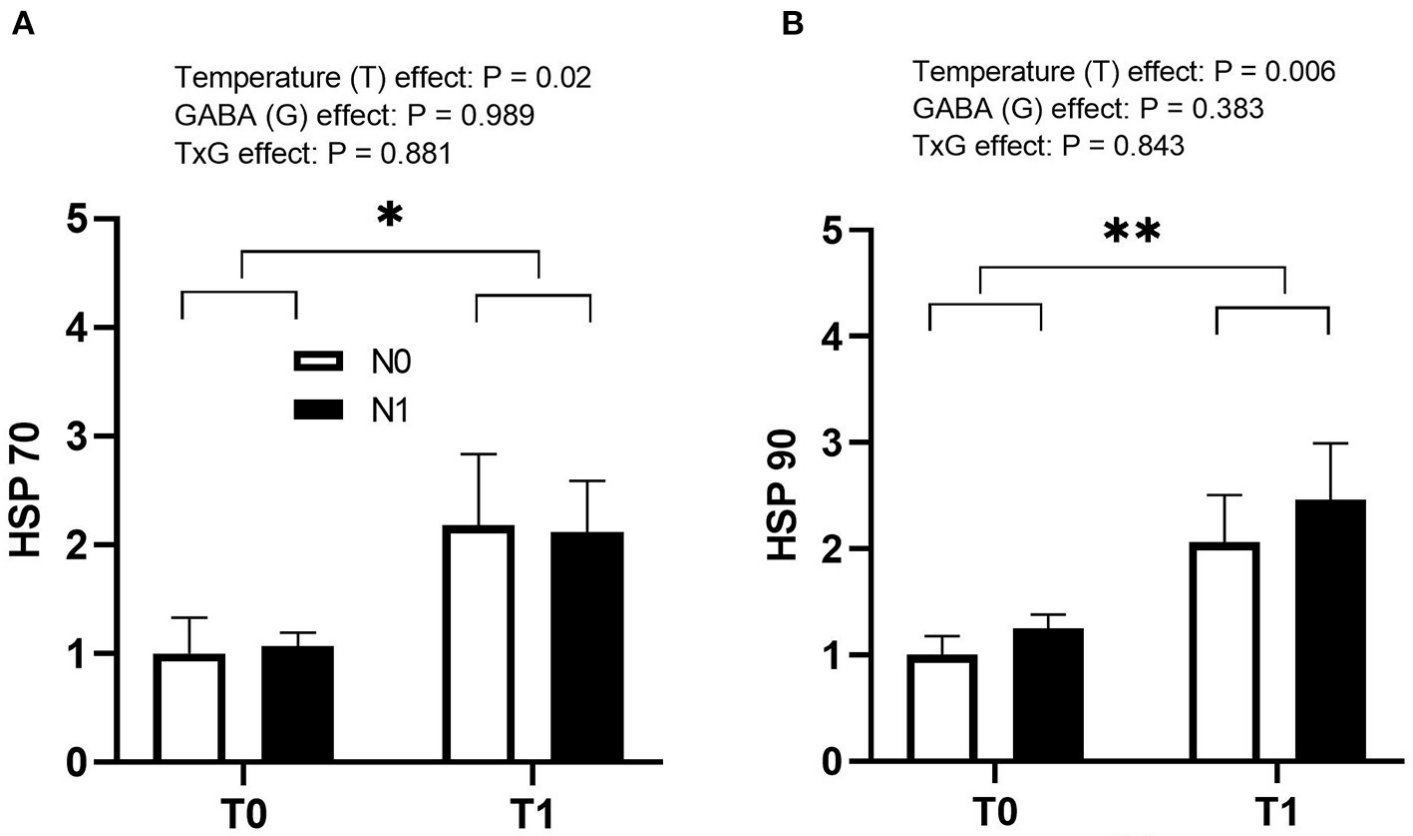
Effects of thermal manipulation (TM) and GABA supplementation (GABA) during embryogenesis on the relative expression of **(A)** HSP70 and **(B)** HSP90 genes at hatch. Data show mean ± SEM (*n* = 6). T0: Normal temperature; T1: thermal manipulation; N0: No *in ovo* injection; N1: *in ovo* GABA injection. ^*^indicates significance at *P* < 0.05. ^**^ indicates significance at *P* < 0.01.

T1 resulted in a significant upregulation of HSP70 (*p* = 0.02) and HSP90 (*p* = 0.006) genes in the liver at the time of hatch (Figure 4). However, there was no significant effect for N1 or interaction between T1 and N1.

**Figure 5:**
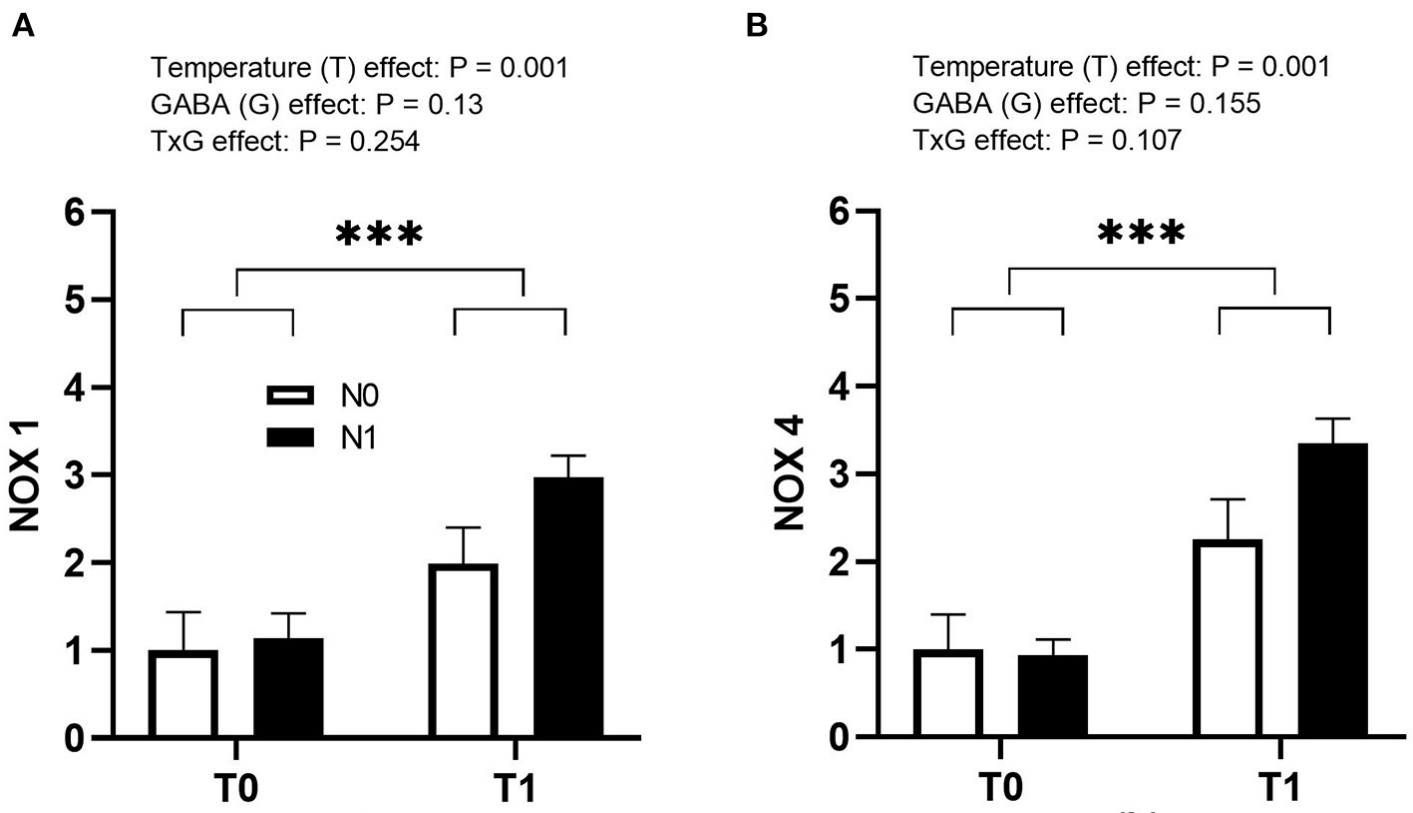
Effects of thermal manipulation (TM) and GABA supplementation (GABA) during embryogenesis on the relative expression of **(A)** NOX1 and **(B)** NOX4 genes in liver at hatch. Data show mean ± SEM (*n* = 6). T0: Normal temperature; T1: thermal manipulation; N0: No *in ovo* injection; N1: *in ovo* GABA injection. ^***^indicates significance at *P* < 0.001.

Both NOX1 and NOX4 genes were significantly increased by T1 during incubation (*p* = 0.001), but N1 or interaction was not significant at the time of hatch (Figure 5).

**Figure 6:**
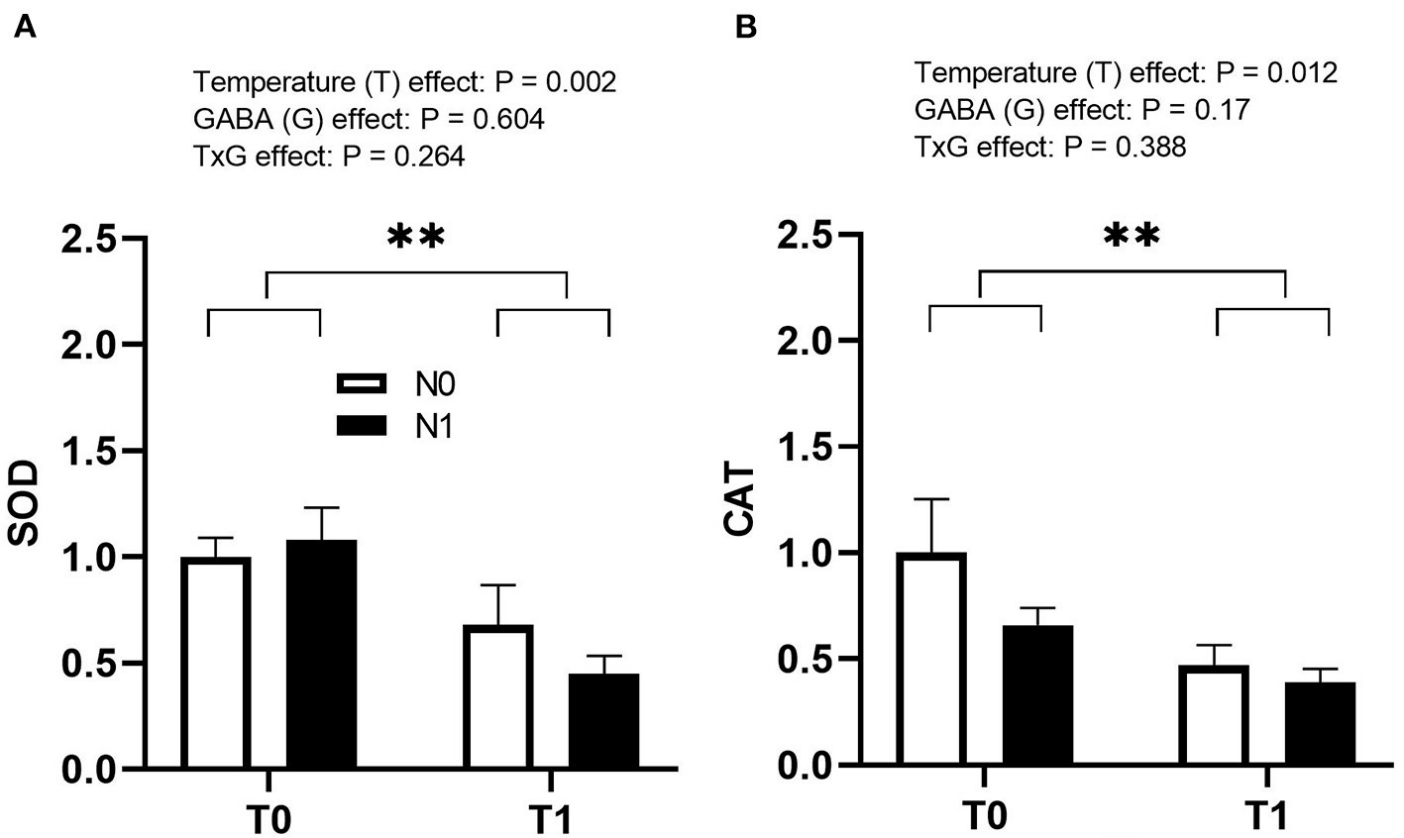
Effects of temperature manipulation (TM) and GABA supplementation (GABA) during embryogenesis on the relative expression of **(A)** SOD and **(B)** CAT genes in liver at hatch. Data show mean ± SEM (*n* = 6). T0: Normal temperature; T1: thermal manipulation; N0: No *in ovo* injection; N1: *in ovo* GABA injection. ^**^indicates significance at *P* < 0.01.

On the other hand, both SOD (*p* = 0.002) and CAT (*p* = 0.012) genes were significantly downregulated in the liver by T1 (Figure 6). N1 did not affect the expression of antioxidant enzyme-related genes, and no significant interaction was observed.

## Discussion

The current study was conducted to examine the impact of embryonic ETM with or without GABA on hatching performance and hepatic gene expression of stress- and antioxidant-related enzymes in newly hatched broiler chicks. Hatchability records show that all the treatment groups including control had hatchability ranging between 69.8 and 77.8%. The reason behind the poor hatchability could be related to the age, nutrition, and environmental conditions of the breeder birds. No significant changes in hatching rates between T0N0 and T0N1 suggest that *in ovo* injection of GABA did not adversely affect the survivability of developing embryos. However, the hatchability of T1N1 decreased slightly compared to T0N0, T0N1, and T1N0. ETM has been considered differently as thermal conditioning (9, 28) or embryonic stress (21, 29). The logic of thermal conditioning can also be seen as the limited stress the embryo can tolerate without showing harmful effects. However, additional handling of the eggs due to *in ovo* GABA supplementation may have resulted in decreasing the hatchability in T1N1. Numerically higher values of BW at hatch in the same group also indicate that weaker embryos could not survive the dual stress (T1N1).

Temperature is an important aspect of artificial incubation and has a significant effect on hatchability parameters such as BW (6). Previous studies have reported inconsistent effects of ETM on BW at hatch, either ineffective (30) or reduced BW at hatch (10). On the other hand, others have concluded that ETM increases BW at hatch (9, 28). The discrepancy in the results could be due to the difference in the temperature, intensity, or duration of ETM. In the current study, the increased BW at the hatch in T1 could be attributed to the development of thermotolerance arising due to daily cyclic variation of 6 h exposure to the higher temperature. Another possibility could be due to the survival of healthier embryos and can be correlated with the lower hatchability in T1N1. Furthermore, *in ovo* feeding of GABA also helped in gaining higher BW at hatch (Table 3). BW regulation involves complex processes including food consumption, digestion, nervous and endocrine systems such as thyroid and growth hormone. The effect of GABA on BW gain might be attributed to improved nutrient utilization through enhanced activity of digestive enzymes and thyroidal stimulation by growth hormone (17, 31).

Although the current study showed a significant interaction in hepatic MDA levels at hatch, neither ETM nor *in ovo* GABA supplementation significantly affected MDA levels. It appears that MDA concentrations depend on the type, severity, and duration of stress, such as heat stress. It has been previously demonstrated that MDA concentrations in serum, plasma, and tissues are much lower in chronic heat stress than in acute heat stress (4) and that there is no difference in hepatic MDA levels after receiving cyclic heat stress for 6 weeks at 32°C for 5 h (32). This suggests that MDA levels increase in the initial phase of stress, but become normal due to adaptation if the stress persists for a long time. In the present study, T0N1 had higher MDA levels than T0N0. The exact reason behind this is not clear. However, there is a possibility that *in ovo* feeding of GABA during embryogenesis may have resulted in increasing the MDA levels in chicks at hatch. Previous reports also suggested that *in ovo* supplementation of nutrients such as vitamin C at 17th ED increases plasma MDA linearly (33). In the present scenario, many research laboratories use mechanical processes for *in ovo* supplementation, which may require extra handling, create disturbance to the embryo, and possibly cause a mild increase in MDA levels. The lack of information on the effects of ETM and *in ovo* feeding on MDA levels in various tissues requires further research to establish the mechanism behind the role of MDA regulation at hatch.

HSPs are major stress-related proteins that have been extensively studied in response to several stressors. Among them are the most explored proteins HSP70 and 90 due to their highly conserved nature (32). It has already been documented that the expression of HSP70 and 90 genes increases under acute or chronic heat stress in chickens during the rearing period (19, 34). Previous studies conducted in our laboratory showed that *in ovo* GABA supplementation helped decrease the expression of HSP70 gene when exposed to acute heat stress (38°C for 3 h) in 10-day-old chicks (12), but did not significantly affect the expression of HSP-related genes when exposed to cyclic heat stress (33°C for 6 h daily) from 28 to 31 days of age (25). However, scanty information is available about the expression of HSP genes in chicks at hatch after ETM and *in ovo* feeding. In the present study, T1 significantly increased the expression of HSP70 and 90 genes (*p* < 0.05) at hatch. These are consistent with previous studies in which ETM at 38.8°C for 6 h or 18 h a day from 10th to 18th ED had higher expression of HSP70 gene during the embryogenesis (35). Furthermore, higher HSP90 gene expression is associated with enhanced survivability of cells grown in an adverse environment (27). Thus, the increased expression of HSP-related genes after ETM may be due to its protective effects on cells. The development of tolerance against stress after ETM could be due to hormonal manifestation or behavioral adjustments (36) associated with the enhanced expression of HSP.

In the present study, the expression of NOX1 and NOX4 genes in the liver increased at hatch when embryos were incubated under high-temperature conditions, indicating higher production and accumulation of ROS in T1 chicks at hatch. NOX is a complex of different isozymes present at the surface of the membrane and acts as an oxygen sensor (37) and acts as a source of ROS production under stress conditions (38). Previous studies reported increased expression of NOX-related genes in avian cells exposed to heat stress at 41°C for 6 h (39). However, higher expression of NOX had no visible adverse effects in the current study. Furthermore, the fact that T1 resulted in enhanced BW along with higher NOX-related gene expression suggests that NOX accumulation at hatch may develop the ability of the chicks to withstand enhanced ROS in the later stages of life. This is evident from the previous studies where ETM showed a long-lasting effect by enhancing the tolerance capacity in 28-day old Cobb broilers by decreasing the hepatic NOX4 gene expression in acute heat-stressed birds exposed at 40°C for 1, 3, 5, and 7 h (7).

The antioxidant system comprises various antioxidant enzymes that activate in response to any kind of stress and prevent the oxidative damage caused by stress. SOD catalyzes the dismutation of superoxide radicals into hydrogen peroxide while CAT functions by catalyzing the breakdown of hydrogen peroxide into water molecules (40). The activity of SOD and CAT depends on the type of stress. For instance, the activity of antioxidant enzymes increases with short-term stress and decreases with long-term exposure (41, 42). ROS production is initiated under thermogenic conditions resulting in oxidative stress to generate excessive free radicals and superoxide (42, 43). In short-term stress, the antioxidant system significantly increases the activity of SOD and CAT, neutralizing free radicals and superoxide, rapidly exerting a protective effect on cells. However, in the present study, ETM was performed for 6 h a day from 10th to 18th ED. This can act as long-term exposure of the embryos to mild stress resulting in decreased gene expression for antioxidant enzymes (CAT and SOD) in TM chicks on the day of hatch. Long-term stress results in the accumulation of ROS due to the disruption of the antioxidant system, thus decreasing the activity of antioxidant enzymes (44). Concomitantly, T1 chicks may have the ability to tolerate higher oxidative stress later in life due to their increased ability to accumulate ROS. As a result, ETM chicks can withstand stress more conveniently (36).

The weight of most of the organs was not affected by ETM or *in ovo* GABA supplementation. This can be seen as a constructive approach to counter treatment (T1 or N1) presenting no harmful effects. Al Wakeel and his group reported the role of dietary GABA in enhancing feed intake and improving gut health (19). In the present study, *in ovo* GABA administration resulted in improving relative intestine weight and BW at hatch. To our best knowledge, this is the first study to evaluate the effect of *in ovo* GABA supplementation at hatch. The higher intestine weight in T0N1 suggests better gut health at hatch. This could be due to the enhanced uptake of nutrients during the last 3 days of incubation after *in ovo* GABA supplementation. In correlation, the treatment effect was also observed in terms of BW, and N1 was found to be effective in increasing the chick's BW at hatch. This confirms the role of GABA in improving growth performance due to better utilization of nutrients during incubation. Elwan and his group previously reported the role of *in ovo* feeding different supplements such as amino acids in improving BW at hatch (29). Furthermore, studies conducted during the rearing period have also reported higher growth and better gut health after dietary GABA supplementation in chickens exposed to stress (13, 19). The present study evaluated only the day of hatch, but a more pronounced effect could have been observed during the mid or after 1 week of age due to the availability of external feed and opens the window for future studies. No further effects were observed in the remaining organ weight and expression of HSP, NOX, and antioxidant enzyme-related genes in this study, suggesting no harmful effects with *in ovo* GABA supplementation.

In conclusion, T1 and N1 independently improved broiler BW at hatch, but T1 strongly regulated stress-related hepatic gene expression. Enhanced expression of HSP and NOX-related genes with increased BW at hatch suggests better health and viability of T1 chicks in adverse environments. GABA's effectiveness in protecting against stress might be elucidated through gut health and may have an indirect effect on the antioxidant enzyme system. Further studies are required to evaluate the effect of *in ovo* GABA feeding on gut health to predict its precise mechanism of action in chicks.

## Data Availability Statement

The datasets presented in this study can be found in online repositories. The names of the repository/repositories and accession number(s) can be found below: GenBank; accession numbers are included in the manuscript/supplementary materials.

## Ethics Statement

The animal study was reviewed and approved by Institutional Animal Care and Use Committee of Gyeongsang National University GNU-200916-C0058.

## Author Contributions

AG, CMN, and Y-HC: conceptualization and writing—review and editing. AG and CMN: methodology and data curation. AG, CMN, and C-MJ: software. AG and Y-HC: validation. AG: writing—original draft preparation. Y-HC: supervision. All authors have read and agreed to the published version of the manuscript.

## Funding

This research was supported by the Brain Pool Program funded by the Ministry of Science and ICT through the National Research Foundation of Korea (2019H1D3A1A01071142).

## Conflict of Interest

The authors declare that the research was conducted in the absence of any commercial or financial relationships that could be construed as a potential conflict of interest.

## Publisher's Note

All claims expressed in this article are solely those of the authors and do not necessarily represent those of their affiliated organizations, or those of the publisher, the editors and the reviewers. Any product that may be evaluated in this article, or claim that may be made by its manufacturer, is not guaranteed or endorsed by the publisher.
